# Types, Aspects, and Impact of Relocation Initiatives Deployed within and between Long-Term Care Facilities: A Scoping Review

**DOI:** 10.3390/ijerph19084739

**Published:** 2022-04-14

**Authors:** Damien S. E. Broekharst, Mara P. J. Brouwers, Annerieke Stoop, Wilco P. Achterberg, Monique A. A. Caljouw

**Affiliations:** 1 Department of Public Health and Primary Care, Leiden University Medical Center, 2300 RC Leiden, The Netherlands; w.p.achterberg@lumc.nl (W.P.A.); m.a.a.caljouw@lumc.nl (M.A.A.C.); 2 University Network for the Care Sector South Holland, Leiden University Medical Center, 2300 RC Leiden, The Netherlands; 3 Department of Health Services Research, Maastricht University, 6200 MD Maastricht, The Netherlands; mpj.brouwers@maastrichtuniversity.nl; 4 Living Lab in Ageing and Long-Term Care, Maastricht University, 6200 MD Maastricht, The Netherlands; 5 Academic Collaborative Center Older Adults, Tranzo, Tilburg University, 5037 AB Tilburg, The Netherlands; h.j.stoop@tilburguniversity.edu

**Keywords:** relocation initiative, transition, relocation, transfer, long-term care, scoping review

## Abstract

Relocation of residents within or between long-term care facilities occurs regularly. To mitigate potential negative consequences, supportive relocation initiatives have been developed. This scoping review addresses types, aspects, and impact of relocation initiatives developed to relocate residents between or within long-term care facilities. A total of 704 articles were identified in a systematic literature search of 11 databases between April and July 2021. Using predefined eligibility criteria, two researchers independently screened titles and abstracts, resulting in 36 articles for full-text screening. Finally, six articles were included. Analysis was performed using thematic coding. Three types of relocation initiatives were identified, namely, interventions (*n* = 3), guidelines (*n* = 2), and a plan (*n* = 1). These initiatives described specific aspects of relocation, namely, spatial orientation (*n* = 3), practical assistance (*n* = 3), psychological support (*n* = 3), staff preparation (*n* = 2), and client engagement (*n* = 2). Only three intervention studies reported the impact of relocation initiatives on residents, namely, improved mental health (*n* = 3), spatial orientation (*n* = 2), self-reliance (*n* = 2), and social behavior (*n* = 1). The scope of the found relocation initiatives was often limited as they focused on specific designs, aspects, and residents. Therefore, the complexity of relocation processes is often overlooked, and more comprehensive relocation initiatives should be developed.

## 1. Introduction

Relocation of long-term care residents within or between long-term care facilities occurs for a variety of reasons, ranging from changing healthcare needs to closure or renovation of long-term care facilities [1,2,3]. Although these relocations could substantially improve the lives of long-term care residents over time [1,2,3], the actual process of relocating within or between long-term care facilities often induces physical discomfort (e.g., headaches, muscle tension, insomnia), as well as psychological problems (e.g., stress, anxiety, depression), in long-term care residents [1,2,3]. In order to prevent, mitigate, or eliminate these negative consequences, many have sought to develop relocation initiatives that support these long-term care residents [1,2,3].

Relocation initiatives include any set of practices, methods, protocols, policies, guidelines, or programs designed to ensure the coordination and continuity of healthcare as individuals or groups of residents are transferred between different locations or different levels of care within the same location [4]. In the current scientific literature, several types of relocation initiatives have been developed to improve the transfer of individuals between their home and a long-term care facility or between a hospital and a long-term care facility [5,6]. These relocation initiatives can be pre-relocation initiatives focused on preparing for new living conditions, post-relocation initiatives aimed at becoming familiar with the new circumstances, or bridging initiatives aimed at safeguarding the quality and continuity of care during the relocation [5,6]. Many of these relocation initiatives have shown to be effective in eliminating stress, preventing loss of control, mitigating depressive disorders, and reducing patient safety incidents [4].

By contrast, it seems that little research has been conducted on the development and effectiveness of relocation initiatives involving the transfer of long-term care residents between or within long-term care facilities [2,7]. As these relocations occur on a daily basis in long-term care facilities, it is necessary to map the existing literature on relocation initiatives to inform professionals in long-term care facilities on the initiatives that are available to them, as well as to provide researchers with insight into the existing literature and possible avenues for future research. Therefore, this scoping review specifically addresses types, aspects, and impact of relocation initiatives deployed to relocate long-term care residents between or within long-term care facilities.

## 2. Materials and Methods

In this scoping review, the methodological steps outlined in the framework of Arksey and O’Malley [8] were followed. This framework includes five steps: (1) identifying the research question, (2) identifying relevant studies, (3) selecting appropriate studies, (4) charting the data, and (5) collating, summarizing, and reporting the results [8].

### 2.1. Identifying the Research Question

The research questions were as follows: (1) What relocation initiatives are used for transitions within and between long-term care facilities? (2) What are the key aspects of these relocation initiatives? (3) What is the impact of these relocation initiatives on long-term care residents?

### 2.2. Identifying Relevant Studies

**Figure 1:**
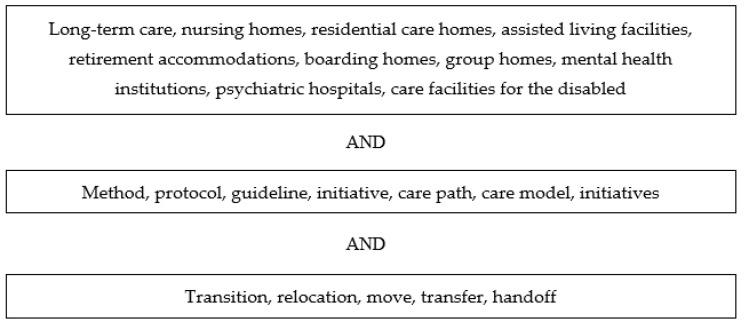
Search terms.

Between April and July 2021, a systematic literature search was conducted in the electronic databases PubMed, Medline, Embase, Web of Science, Cochrane Library, Emcare, PsycINFO, PsycArticles, Psychology and Behavioral Sciences Collection, Academic Search Premier, Social Services Abstracts, and Sociological Abstracts. These databases were searched for scientific papers describing relocation initiatives regarding the transfer of long-term care residents within and between long-term care facilities. Scientific papers in all languages were included, and no specific time period was selected in order to make the literature search as comprehensive as possible. Figure 1 shows the search terms that were used to search the titles and/or abstracts of potentially relevant papers.

### 2.3. Selecting Appropriate Studies

Two researchers (D.B. and M.B.) independently checked the papers yielded by the systematic literature search for eligibility by screening their titles and abstracts. For the present study, papers were eligible if they met the criteria in Table 1. When considered eligible by both reviewers, the full-text paper was retrieved and again reviewed for eligibility again, with the same eligibility criteria. Any disagreement between the reviewers was resolved by consulting a third researcher (M.C.).

Duplicate studies, non-retrievable full-text papers, and nonscientific papers (e.g., editorials) were excluded. For this study, the methodological rigor of the included studies was not evaluated [8]. This was in line with the principles of scoping reviews, as the focus of our study was to map the existing literature on a specific topic (in this case, relocation initiatives used for transitions in and between long-term care facilities).

### 2.4. Charting the Data

Two authors (D.B. and M.B.) extracted relevant data from the selected studies. A pre-formulated data extraction sheet was developed to capture all relevant aspects. The identified relocation initiatives were reviewed in terms of key aspects (i.e., authors, country, type of initiative, objective of initiative, professionals involved, content of initiative, target group) and were analyzed using thematic coding. The decision to focus on this information was made after extensive review of the included studies to determine the most important features of the selected papers and a subsequent discussion between all authors.

### 2.5. Collating, Summarizing, and Reporting the Results

The findings of this scoping review are displayed in figures and tables to systematically present all specific details about the types and aspects of relocation initiatives found in the existing scientific literature, as well as in a narrative format to describe their impact on relevant stakeholders. The results of this scoping review are reported according to the PRISMA scoping framework [9].

## 3. Results

### 3.1. Study Retrieval

**Figure 2:**
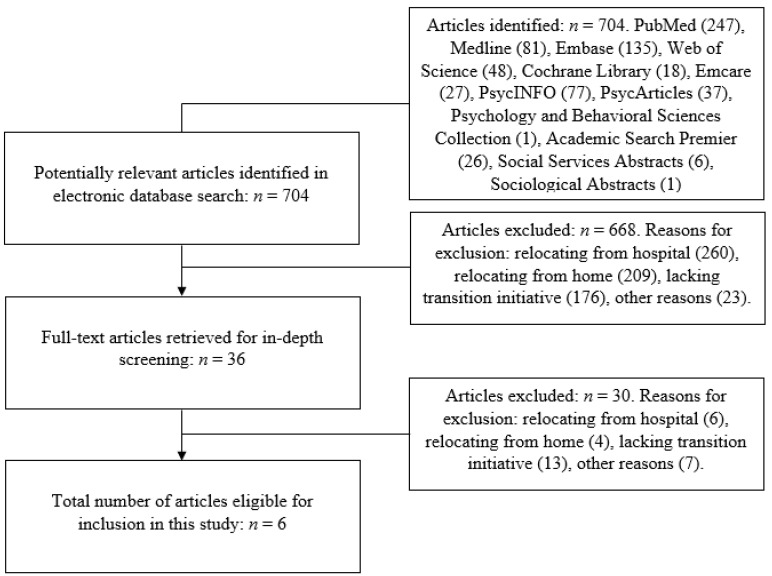
Flow diagram.

A total of 704 articles were identified (Figure 2). After assessing the titles and abstracts of these articles according to the eligibility criteria in Table 2, 36 articles remained for full-text screening. This screening resulted in six articles suitable for inclusion in the scoping review.

All six articles described relocation initiatives in Western countries. Five targeted a specific set of aspects of the relocation process, while one took a more comprehensive approach [10]. Five articles described relocation initiatives directed at nursing home residents, while one described a relocation initiative directed at intellectually disabled persons [11]. The three articles that evaluated the impact of relocation initiatives exclusively examined the impact on residents in terms of, for instance, mental health, spatial orientation, self-reliance, and social behavior [12,13,14].

**Table 2:** Extraction table.

Authors	Country	Type of Transition Initiative	Objective of Transition Initiative	Professionals Involved	Key Aspects	Target Group
McGilton et al. (2003) [13]	Canada	Intervention	Way-finding initiative used to make relocated nursing home residents aware of their surroundings	Primary nurses	The initiative uses landmarks as environmental cues, and provides the residents opportunities to learn and/or relearn a routine set of behaviors	Nursing home residents with dementia involved in relocation between long-term care facilities (treatment group: *n* = 17, 94% female, 6% male, mean age 86.2; control group: *n* = 15, 67% female, 33% male, mean age 89.2)
Bekhet et al. (2016) [12]	United states	Intervention	Resourcefulness training initiative used to improve resilience of relocated nursing home residents	Nurse clinician trained in the initiative	The initiative consisted of 6 sessions lasting 1.5 h in which older adults learn skills regarding coping strategies, problem solving, positive self-talk, priority setting, and decision making	Nursing home residents involved in relocation between long-term care facilities (total group: *n* = 38, 75% female, 25% male, mean age 78.0)
Nirenberg (1983) [14]	United States	Intervention	Preparatory relocation initiative used to get both high-functioning and low-functioning nursing home residents ready for relocation	Nurses and volunteers	The initiative consisted of a pre-visit to the new facility, a few meetings with a volunteer from the community, a slide presentation about the relocation, frequent reminders about the move, and some involvement in (un)packing	Nursing home residents involved in relocation between long-term care facilities (high-functioning group: *n* = 20, 50% female, 50% male, mean age 60.2; low-functioning group: *n* = 20, 45% female, 55% male, mean age 61.8)
Wullink et al. (2007) [11]	Netherlands	Guideline	Transfer of medical care guideline used to manage the transfer of medical care from intellectual disability specialist physicians to general practitioners	Intellectual disability specialist physicians, general practitioners	The guideline consists of four elements concerning handling and storage of patient information, handling of out-of-hours calls, visits and referral, and appropriate communications between support staff and professionals	Intellectually disabled people involved in relocation between long-term care facilities
Hertz et al. (2016) [15]	United States	Guideline	Evidence-based guideline used to manage the relocation of cognitively intact older adults	Nurses, physicians, social workers, case managers, discharge planners, family members	The guideline consists of two elements concerning the pre-relocation and post-relocation assessment of needs and risks	Cognitively intact older adults involved in relocation between long-term care facilities
Grant (1997) [10]	Canada	Relocation plan	Relocation plan used to organize the relocation of nursing home residents	Senior management	The relocation plan has three objectives concerning involvement of residents, minimizing stress, and preparing the staff	Senior management involved in relocation between long-term care facilities

### 3.2. Types of Relocation Initiatives

Three types of relocation initiatives were found, namely, (1) relocation interventions, (2) relocation guidelines, and (3) relocation plans.

#### 3.2.1. Relocation Intervention

In three articles, relocation interventions were outlined [12,13,14]. These relocation interventions consisted of a deliberate and specified set of actions (e.g., using landmarks as environmental cues in order to make residents aware of their surroundings), implemented to improve the relocation of residents between or within long-term care facilities.

#### 3.2.2. Relocation Guidelines

Two articles described relocation guidelines [11,15]. These relocation guidelines described certain general principles (e.g., the person responsible for finding a general practitioner should make arrangements with the general practitioner about out-of-hours house calls and practice visits) that should be adhered to in the relocation of residents between or within long-term care facilities.

#### 3.2.3. Relocation Plans

One of the articles reported on a relocation plan [10]. This relocation plan was a specified roadmap outlining successive steps and objectives (e.g., the first objective focused on giving residents a say in planning their move and designing their room) that need to be taken to complete the relocation of residents between or within long-term care facilities.

### 3.3. Aspects of Relocation Initiatives

Five aspects of relocation initiatives were found, namely, (1) spatial orientation, (2) practical assistance, (3) psychological support, (4) staff preparation, and (5) client engagement.

#### 3.3.1. Spatial Orientation

Three articles emphasized spatial orientation [10,13,14]. Grant [10] established a relocation plan in which increasing residents’ familiarity with the new facility prior to the move and improving the spatial orientation of residents in the new facility following the move were emphasized. McGilton et al. [13] developed a relocation intervention in which residents were made aware of their surroundings after their relocation by using landmarks, and by providing opportunities to (re)learn a routine set of behaviors. Nirenberg [14] designed a relocation intervention in which pre-visits to the new facility were crucial for increasing residents’ familiarity with their new surroundings.

#### 3.3.2. Practical Assistance

In three articles, the relocation initiative focused on practical assistance [10,14,15]. Grant [10] developed a relocation plan in which providing residents with practical assistance during the relocation to the new facility was considered crucial. Hertz et al. [15] designed relocation guidelines in which assistance with identifying the need to move, locating a new residence, and preparing for the move were described. Nirenberg [14] established a relocation intervention which partially focused on assistance with (un)packing the belongings of residents.

#### 3.3.3. Psychological Support

Three articles stressed psychological support as an important aspect of relocation initiatives [10,12,15]. Bekhet et al. [12] developed a relocation intervention in which older adults learned coping strategies, problem solving, positive self-talk, priority setting, and decision making to improve resilience before, during, and after the relocation process. Grant [10] established a relocation plan in which minimizing disruption to familiar routines and programming prior to, during, and after the move were emphasized. Hertz et al. [15] reported relocation guidelines in which psychological support for individuals at risk of maladjustment was important.

#### 3.3.4. Staff Preparation

Two articles emphasized staff preparation as a key aspect of relocation initiatives [10,11]. Grant [10] established a relocation plan in which educating staff about stressful effects of a move, providing staff with opportunities to address problems, familiarizing staff with the new facility, and allowing staff to express their concerns played an important role. Wullink et al. [11] developed relocation guidelines emphasizing sound practice regarding handling and storage of medical information, handling of out-of-hours calls, visits and referral, and appropriate communications between support staff and professionals.

#### 3.3.5. Client Engagement

In two articles, the relocation initiatives emphasized client engagement [10,14]. Grant [10] established a relocation plan in which residents were involved in the planning process and given control over some aspects of the move. Nirenberg [14] developed a relocation intervention in which meetings with a volunteer from the community, a slide presentation about the relocation, and frequent reminders about the move played an important role.

### 3.4. Impact of Relocation Initiatives

Only three articles about relocation interventions described the impact of relocation on long-term care residents [12,13,14]. These articles did not consider the impact on healthcare professionals, managers, family, or other stakeholders. Impact was measured and reported with regard to (1) mental health, (2) spatial orientation, (3) self-reliance, and (4) social behavior.

#### 3.4.1. Mental Health

In three articles, the impact on mental health of long-term care residents was evaluated [12,13,14]. Bekhet et al. [12] show that residents participating in the relocation intervention experienced a slight but nonsignificant increase in the mean scores of positive cognition, relocation adjustment, and adaptive functioning compared to the control group after relocation. McGilton et al. [13] suggested that residents involved in the relocation intervention experienced a significant decrease in their level of agitation in comparison to the control group after relocation. Nirenberg [14] found that residents participating in the relocation intervention experienced no significant improvement in their mental state or morale compared to the control group after relocation.

#### 3.4.2. Spatial Orientation

In two articles, the impact concerning spatial orientation of long-term care residents was outlined [13,14]. McGilton et al. [13] found that residents involved in the relocation intervention experienced a significant increase in their ability to find specific locations, such as the dining room, relative to the control group after relocation. Nirenberg [14] showed that residents participating in the relocation intervention experienced significant improvement in their face–hand coordination compared to the control group after relocation.

#### 3.4.3. Self-Reliance

Two articles described the impact on self-reliance of long-term care residents [12,14]. Bekhet et al. [12] showed that residents involved in the relocation intervention experienced a slight but nonsignificant increase in the mean score of personal resourcefulness, social resourcefulness, and total resourcefulness relative to the control group after relocation. Nirenberg [14] indicated that residents participating in the relocation intervention experienced significant improvement in grooming, cleaning, and consuming compared to the control group after relocation.

#### 3.4.4. Social Behavior

One article reported on the impact on social behavior of long-term care residents [14]. Nirenberg [14] found that nursing home residents participating in the relocation intervention experienced significant improvement in social activity, interaction behavior, and proximity to others compared to the control group after relocation.

## 4. Discussion

This study described a comprehensive scoping review of the scientific research on relocation initiatives for the relocation of residents within or between long-term care facilities. It specifically addressed types, aspects, and impact of these relocation initiatives. Three types of initiatives were found, namely, relocation interventions, relocation guidelines, and relocation plans. Important aspects in these initiatives were spatial orientation, practical assistance, psychological support, staff preparation, and client engagement. Only the interventions examined the impact of relocation on residents. They reported improved mental health, spatial orientation, self-reliance, and social behavior.

This study reveals that most articles found on the relocation of residents between or within long-term care facilities described relocation interventions, which is in line with previous research [16,17,18,19]. This study also suggests that there are more types of relocation initiatives, such as relocation guidelines and relocation plans. A possible explanation for this intervention focus is that relocation interventions are easier to evaluate using scientific methods than the more practically relevant relocation guidelines and relocation plans.

The results suggest that practical assistance, psychological support, client engagement, and staff preparation are important aspects of relocation initiatives deployed for the relocation of residents between or within long-term care facilities, which is congruent with prior studies [20,21]. However, the results also indicate that spatial orientation is an important aspect. One might suggest that spatial orientation is emphasized more strongly in these relocation initiatives as residents often have to adjust to new and innovative living concepts that differ from their familiar surroundings [13].

The findings also indicate that using relocation initiatives to transfer residents within or between long-term care facilities positively impacts their mental health, self-reliance, and social behavior, which is in accordance with previous studies [22]. However, the findings also show that using these relocation initiatives positively impacts spatial orientation in residents. This improved spatial orientation may be explained by the emphasis these relocation initiatives place on the familiarization of residents with their new surroundings [13].

In addition to these results, four other findings are important to discuss. First, this study only identified relocation initiatives in Western countries. A probable explanation is that non-Western countries have healthcare systems and cultures that cause recipients of long-term care to live at home or with family longer, making relocation within or between long-term care facilities less common [23]. Second, the results showed five relocation initiatives regarding the relocation of nursing home residents and only one concerning the relocation of intellectually disabled individuals. This particular emphasis may be explained by the relatively large health risks associated with the relocation of nursing home residents [3]. Third, the impact of the identified relocation interventions was only considered for long-term care residents. However, no impact was found of relocation initiatives on healthcare professionals and family members. This priority for residents may be caused by the fact that they are the primary recipients and subjects of relocation initiatives. Fourth, the findings show that five relocation initiatives were specialized and targeted at improving individual aspect of the relocation process. Only one relocation initiative provided a more comprehensive approach to the relocation process. A probable explanation is that most authors approached the relocation process from their own fields of expertise.

### 4.1. Strengths and Limitations

One strength of this study is that a broad spectrum of databases was included in the systematic literature search. Therefore, a comprehensive search of the existing scientific literature could be performed generating articles from many different scientific fields. Another strength of this study is that articles on a broad spectrum of long-term care residents were included. Therefore, articles with regard to nursing home residents, as well as intellectually disabled persons, were generated. A limitation of this study is that only the scientific literature was included; therefore, the relocation initiatives described in the gray literature were missed.

### 4.2. Practical Implications

This study has several implications for practice. The findings can be utilized by relevant professionals in long-term care facilities to learn about and select the most appropriate type of relocation initiative (e.g., relocation interventions, relocation guidelines, relocation plans) for their specific situation. The results can also be particularly useful to these professionals in the development of comprehensive relocation initiatives as this study provides a wide array of different key aspects (e.g., spatial orientation, practical assistance, psychological support, staff preparation, client engagement) that should be taken into account. The findings further suggest indicators (e.g., mental health, spatial orientation, self-reliance, social behavior) that can be used by these professionals to assess and evaluate the impact of the relocation initiatives they are developing and implementing.

### 4.3. Future Research

This study indicates three avenues for future research. First, it could be beneficial to investigate the existence, suitability, and effectiveness of other important aspects of relocation initiatives (e.g., family involvement) in addition to the aspects found in this study. Second, it could be useful to also investigate the impact of relocation initiatives on relevant stakeholders other than long-term care residents, as the articles identified in this study largely overlooked them. Third, research on more comprehensive relocation initiatives that address and combine multiple aspects of relocation processes is recommended, as the current scientific literature favors the development of specialized relocation initiatives.

## 5. Conclusions

Given the results of this study, it is evident that the existing scientific literature on relocation initiatives deployed for the relocation of residents within or between long-term care facilities is scarce. The scope of the relocation initiatives found was also fairly limited, as they often focused on specific types, aspects, and residents. Due to this limited scope of relocation initiatives, the complex and multifaceted nature of relocation processes was overlooked. This complex and multifaceted nature of relocation processes requires the development and implementation of comprehensive relocation initiatives that take into consideration a wide array of types, aspects, and residents.

## Figures and Tables

**Table 1:** Eligibility criteria.

Eligibility Criteria	
Type of study	Human study
Type of reference	Scientific literature consisting of original research articles, systematic reviews, meta-analyses, narrative reviews, and scoping reviews irrespective of design.
Type of subjects	Adults living in long-term care facilities (i.e., nursing homes, residential care homes, assisted living facilities, retirement accommodations, group homes, mental health institutions, care facilities for the disabled, continuing care retirement communities).
Type of initiative	Methods, protocols, guidelines, interventions, care paths, care models, programs, relocation plans, and best practices established to plan and organize the relocation of individuals within and between long-term care facilities.
Type of facility	A variety of care and support services which help to meet both the medical and the nonmedical needs of adults suffering from old age, chronic illness, or disability, who cannot care for themselves for extended periods.
Language	All languages.
Period of time	No specific period of time.

## Data Availability

Not applicable.
